# Glucocorticoid-Induced Leucine Zipper: A Promising Marker for Monitoring and Treating Sepsis

**DOI:** 10.3389/fimmu.2020.606649

**Published:** 2020-12-16

**Authors:** Ya-Jun He, Ji-Qian Xu, Miao-Miao Sun, Xiang-Zhi Fang, Zhe-Kang Peng, Shang-Wen Pan, Ting Zhou, Ya-Xin Wang, You Shang

**Affiliations:** ^1^ Department of Critical Care Medicine, Union Hospital, Tongji Medical College, Huazhong University of Science and Technology, Wuhan, China; ^2^ Institute of Anesthesiology and Critical Care Medicine, Union Hospital, Tongji Medical College, Huazhong University of Science and Technology, Wuhan, China

**Keywords:** sepsis, glucocorticoid-induced leucine zipper, glucocorticoids, sepsis-induced immunosuppression, anti-inflammatory

## Abstract

Sepsis is a clinical syndrome that resulting from a dysregulated inflammatory response to infection that leads to organ dysfunction. The dysregulated inflammatory response transitions from a hyper-inflammatory phase to a hypo-inflammatory or immunosuppressive phase. Currently, no phase-specific molecular-based therapies are available for monitoring the complex immune response and treating sepsis due to individual variations in the timing and overlap of the dysregulated immune response in most patients. Glucocorticoid-induced leucine zipper (GILZ), is broadly present in multiple tissues and circumvent glucocorticoid resistance (GCR) or unwanted side effects. Recently, the characteristics of GILZ downregulation during acute hyperinflammation and GILZ upregulation during the immunosuppressive phase in various inflammatory diseases have been well documented, and the protective effects of GILZ have gained attention in the field of sepsis. However, whether GILZ could be a promising candidate biomarker for monitoring and treating septic patients remains unknown. Here, we discuss the effect of GILZ in sepsis and sepsis-induced immunosuppression.

## Introduction

Sepsis is a complex disease that causes life-threatening organ dysfunction due to uncontrolled infection (1). The global burden of sepsis is estimated to be 30 million patient episodes, with a mortality rate approaching 30%–50% annually. Notably, most data are derived from the developed countries, and the true global burden of sepsis is much greater than suggested by these figures (2, 3). The World Health Organization (WHO) rendered sepsis a global health priority in 2017 to improve prevention, diagnosis, and management. Concomitant with early hemodynamic and respiratory support and appropriate antibiotic administration, corticosteroids have been widely used for the management of sepsis as adjuvant therapy to control the immune response to invading pathogens.

Glucocorticoids (GCs) constitute are a class of corticosteroids widely used clinically as anti-inflammatory and anti-shock drugs. The therapeutic glucocorticoids include hydrocortisone, prednisolone and dexamethasone. Several studies and meta-analyses have indicated that low-dose hydrocortisone reduced mortality in patients with septic shock in the intensive care unit (ICU). However, the recent adjunctive corticosteroid treatment in critically ill patients with septic shock (ADRENAL) trial did not show a significantly reduced risk of mortality (4). Mortality is likely associated with the timing of treatment and multiple side effects due to the wide spectrum of effects induced by glucocorticoids (GCs) in addition to the complicated immunopathology of sepsis (5). GCs bind the GC receptor (GR), which belongs to the nuclear receptor superfamily of transcription factors to exert their broad physiological and therapeutic effects.

Glucocorticoid-induced leucine zipper (GILZ or TSC22D3), which is a GC-inducible molecule, has emerged as a GC mediator due to its anti-inflammatory effects and theoretically lacks the side effects of GCs (6). Recently, the potential effect of GILZ in sepsis has gained attention, and GILZ has been recognized as a more promising therapy for polymicrobial sepsis than GCs. Hence, notably, that GILZ may be a checkpoint or even a biomarker of innovative therapies for sepsis-mediated immune responses and is regarded as an actionable target. Here, we review the specific role of GILZ in sepsis.

## Immunopathology of Sepsis

Sepsis induces a complex immune response and this excessive pro-inflammatory response easily elicits the dysregulation of signaling pathways, resulting in tissue damage and organ dysfunction, and induces an immunosuppressive environment, which could increase susceptibility to secondary infections associated with poor outcomes and subsequent mortality (7), although immunosuppressive responses occur simultaneously upon the initiation of innate immune response (8–10).

Increasing evidence suggests that immune cell apoptosis, autophagy, broad metabolic defects, endotoxin tolerance, T cell exhaustion and epigenetic regulation are all contributors, and aerobic glycolysis is crucial for maintaining the function of the immune system (11). For instance, immune cells, including monocytes and T cells, undergo metabolic reprogramming and shift from oxidative phosphorylation (OXPHOS) to aerobic glycolysis when the host is infected (11). Both glycolysis and oxidative metabolism are apparently defective in leukocytes during sepsis-induced immunosuppression. The phosphorylation of extracellular signal regulated kinase (ERK) promotes B-cell death in sepsis (12). The delayed state of neutrophil apoptosis is the most significant change in innate immunity and contributes to the deficits in of bacterial eradication and ongoing continuous dysfunction (13). In addition, a deficit in the capacity to activate nuclear factor-κB (NF-κB) or deacetylation of p65 is closely related to the macrophage reprogramming (14, 15) and sepsis-induced immunosuppression (16).

Identifying treatment and diagnosis guidelines is essential for survival during sepsis. A rich profile of transcriptional shifts occurs in leukocytes within a few hours of exposure to endotoxins (17). Patients with multiple traumas exhibit an immunosuppressed state with large quantities of danger-associated molecular patterns shortly after trauma (18). Similarly, a reduction HLA-DR in monocytes occurs early and is thought to be a marker of an altered immune state (19). Multiple studies exploring sepsis-associated survival also focused on this immunopathology. For instance, studies have focused on antiapoptotic strategies that could reduce mortality in septic mice (20), and immunomodulation with thymosin α1, IFN-γ, IL-7, GM-CSF, interferon-g and the immune checkpoint inhibitor PD-1 in the clinic (11). However, the timing of treatment and side effects associated with suppressing excessive inflammation or enhancing host immunity have not been fully elucidated. Consequently, more timely, suitable and precise therapy is necessary to reduce cell and tissue damage in sepsis.

## Sepsis and Glucocorticoids/Endogenous Glucocorticoids

In 1976, William Schumer’s study showed that septic shock patients could benefit from glucocorticoid drugs, and thus, GCs have attracted much interest in the treatment of sepsis (21). Glucocorticoids represent a type of steroid hormones secreted by the renicapsule that play key roles in the regulation of reproduction, metabolism, and immunization by binding the GR. To date, more than 37 randomized clinical trials have investigated the treatment effect of steroids in sepsis. While steroid administration reverses shock in some sepsis patients, which patients could benefit from this treatment remains unclear (22, 23). The European Society of Intensive Care Medicine and the Society of Critical Care Medicine suggest that some benefit of using corticosteroids in sepsis occurs only if shock is present (24). Recently, the APPROCHSS trial involving 1241 patients evaluated the effect of hydrocortisone plus fludrocortisone therapy and revealed a lower all-cause 90-day mortality and higher vasopressor-free days in the hydrocortisone plus fludrocortisone group than the placebo group (25). However, the ADRENAL trial, which included 3800 patients, showed that septic patients who were treated with low-dose hydrocortisone did not exhibit reductions in 90-day mortality compared with the patients in the placebo group (4). Given the number of cases included, the difference of these two large-scale trials is likely associated with their broad physiological molecular mechanisms of GCs and sepsis.

Actually, sepsis also involves in neuroendocrine mechanisms (26). The neuroendocrine system is triggered once the body is infected or traumatized to restore dynamic balance and fight noxious stimulation, thus to promote tolerance (27). Cortisol, which is a product of neuroendocrine signaling and a glucocorticoid, is the executor of neuroendocrine responses. The high level of plasma cortisol concentrations may be associated with the severity of sepsis because cortisol is not only an essential response for survival but also mediates endotoxin tolerance (28, 29). Furthermore, the molecules that mediates cortisol clearance including the corticosteroid-binding globulin (CBG), cortisol carrier albumin, A-ring reductases, and 11b-hydroxysteroid dehydrogenase type 2(11β-HSD2), are reduced during in early phase of sepsis (30–32). Notably, when was inhibited by cortisol generation with hypophysectomy, the mortality of lipopolysaccharide (LPS)-induced septic shock was significant increased (27). Thus, functioning endogenous GCs are very important for the regulation of the immune mechanism of sepsis.

**Figure 1:**
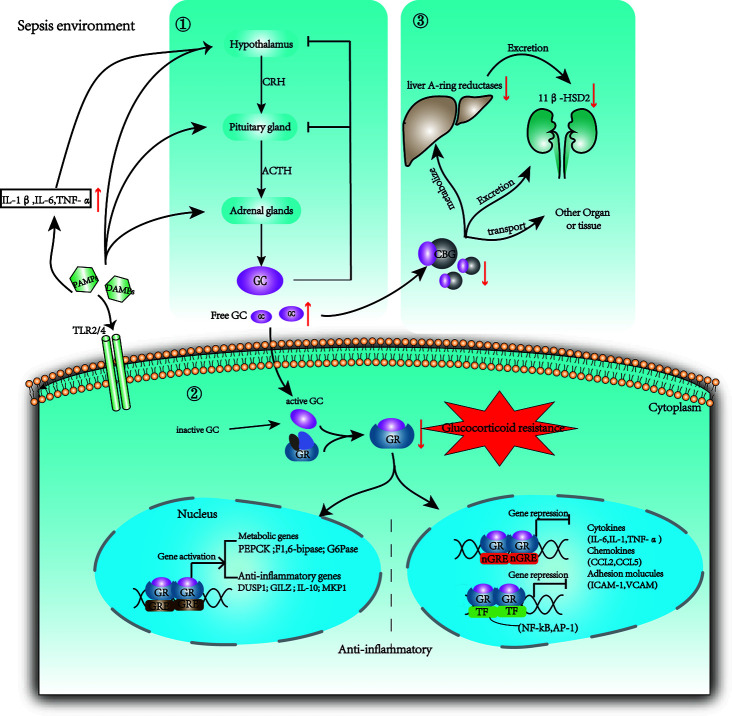
Changes in glucocorticoid production, metabolism, and regulation in sepsis. ① The HPA axis is activated by stress (both physically and mentally), tissue damage, and infection. The paraventricular nucleus of the hypothalamus secretes CRH, and the anterior pituitary gland secretes ACTH or corticotropin; then GC is secreted by the adrenal cortex. Cytokines such as IL-1β, TNF-α, and IL-6 can be projected in the hypothalamus through neuroafferent projections. DAMPs and PAMPs can also directly stimulate adrenocortical cells with toll receptors (TLR), leading to the synthesis of ACTH dependent cortisol. Circulating inflammatory mediators break the brain barrier and act on the hypothalamus. GC exerts negative feedback on both CRH and ACTH production when GC exceeds the threshold. ② Then 5% GC that is free and activated binds the GR and enters the nucleus to influence gene expression. The inactive GC could be reactivated by 11b-HSD1. GR binds promoters to promote metabolic genes and anti-inflammation genes. Additionally, GR binds negative GRE elements (nGRE) on DNA or some transcription factors (TF) to inhibit the expression of target genes. ③75% GC bound to CBG is transported to various tissue. During sepsis, the host protein production is reduced and the CBG in the inflammatory site is cleaved by neutrophil elastases. Cortisol is cleared mainly through A-ring reductases in the liver and through 11β-HSD2 in the kidneys. These enzymes are limited in sepsis resulting in reduced clearance of GC. PAMPs: pathogen-associated molecular patterns; DAMPs: damage-associated molecular patterns; HPA: hypothalamic–pituitary–adrenal axis; CRH: corticotropin-releasing hormone; ACTH: adrenocorticotropic hormone; CBG: corticosteroid-binding globulin; 11β-HSD2: 11b-hydroxysteroid dehydrogenase type 2; 11b-HSD1: 11b-hydroxysteroid dehydrogenase type 1.

However, inadequate cellular corticosteroid activity has been described as critical illness-related corticosteroid insufficiency (CIRCI) and manifests as insufficient glucocorticoid receptor (GR)-mediated downregulation of proinflammatory transcription factors (33). The following three major pathophysiological theories associated with CIRCI: dysregulation of the hypothalamic–pituitary–adrenal (HPA) axis, altered cortisol metabolism (33) and glucocorticoid resistance (GCR), which limits the activity of endogenous GCs’ and GCs’ therapeutic effects (34) (**Figure 1**).

The GR is encoded by a single genetic locus, but alternative splicing of the gene product generates the following four distinct messenger RNAs (mRNAs): GRα, GRβ, GRγ, and GR-A. Each of these intracytoplasmic GRα subtypes can bind GCs and, when in the dimer form, can activate gene expression. The GRα protein normally resides in the cytoplasm, and GRα isoforms directly bind GC response elements (GREs) to induce the transcription of multiple genes, including IL-10, IL-1 receptor antagonists, and GILZ (35). GRα expression significantly decreases in patients suffering sepsis, while GRβ is upregulated and cannot bind GCs in septic patients (36). GRβ located in the nucleus could be induced by several proinflammatory cytokines, such as IL-2, IL-4, IL-17A, IL-17F, IL-23, and TNF-α (37, 38),and is thought to be a negative regulator of GC activity. The overexpression of GRβ inhibits GRα-mediated gene transcription (39, 40). Thus, clearly, high GRβ levels seem to be associated with GCR in sepsis.

There are other forms of GRs, leading to different variants of GR proteins in addition to GRα and GRβ. GR isoforms and associated subtypes within organs may explain sepsis-induced alterations in GC responses, revealing different clinical responses in septic patients (41). Investigators have identified 27 splice variants of the GR gene and hundreds of single nucleotide polymorphisms (SNPs), insertions and deletions, which could lead to different variants of GR proteins (42). Recently, ANP32E has been shown to correlate with GCR. This protein has been linked to the exchange of H2A. z histone and promotes GR-induced transcription (43). The mechanisms of GC-mediated hypo-responsiveness are heterogeneous due to the various cell types and cytokines involved, and further studies of GR biology may be an important step in promoting GC-based therapies. Hence, supplementing active GCs and activating downstream target molecules are critical for this treatment. Consequently, the identification of a potential treatment that can replace GCs and mediate effective anti-inflammatory effects and endotoxin tolerance without causing GC-associated adverse effects has become the main focus of current studies.

## Anti-Inflammatory/Immunosuppressive Effects of Glucocorticoids and GILZ

**Figure 2:**
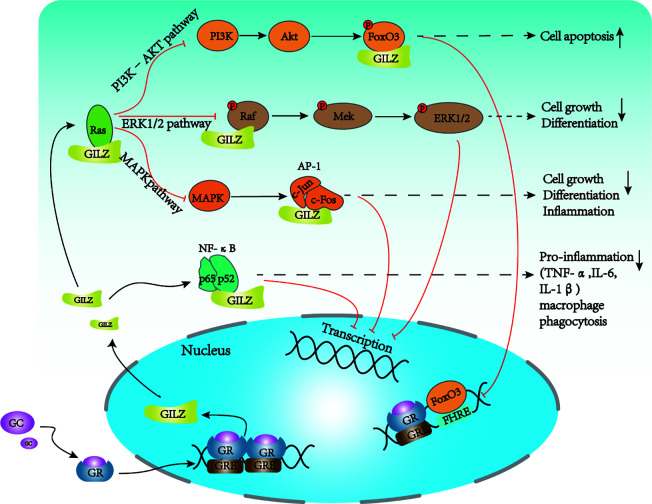
Major signaling pathways of GILZ. GILZ can regulate cell activation, apoptosis, proliferation, and inflammation mainly through several signaling pathways. GILZ directly binds p65 and p52 to inhibit NF-κB signaling to reduce the production of proinflammatory factors and macrophage phagocytosis. GILZ can directly bind Ras and inhibit downstream pathways. (1) inhibits the PI3K–Akt pathway to regulate apoptosis and cell survival; GILZ inhibits FOXO3A-mediated transcription, such as the pro-apoptotic protein, Bim. (2) The direct binding of GILZ to Ras and Raf leads to reduced activation of MEK, ERK, and MAPK, which inhibits cell growth and proliferation. (3) GILZ can directly bind c-Fos and c-Jun to inhibit AP-1 signaling, which prevents cell growth, cell differentiation, and inflammation. NF-κB, nuclear factor κB; AP-1, activator protein 1; FoxO3, forkhead box protein O3; ERK, extracellular signal-regulated kinase; MAPK, mitogen-activated protein kinase; PI3K, phosphoinositide 3-kinase; AKT: Protein kinase B (PKB), also known as Akt.

Glucocorticoids function at physiological and pharmacological levels and are mediated by the GR. Following glucocorticoid exposure, the GC/GR complex translocates into the nucleus and directly binds to glucocorticoid response elements (GREs) to promote downstream transcription factors, including GILZ (44). In turn, GILZ binds NF-κB and prevents its nuclear translocation to elicit anti-inflammatory effects. GILZ can also directly bind c-Jun and c-Fos, which are two constituents of AP-1, to inhibit their transcriptional activity and gene expression of proinflammatory molecules (**Figure 2**). Additionally, GILZ interacts directly with Ras and Raf, thereby inhibiting the downstream activation of mitogen-activated protein kinase 1 (MAPK1), to mediate antiproliferative effects (45, 46).

GILZ, which is widely expressed in various human and mouse organs and is most highly expressed in the lungs (47, 48), was originally identified in 1997 (49). GILZ contains the following three main regions: an N-terminal domain (1–75 amino acids) with a tuberous sclerosis complex (TSC) domain, a leucine zipper (76–97 amino acids) that largely mediates the homodimerization of GILZ, and a proline-rich C-terminal domain (98–137 amino acids) and at least three typical GREs (50) at transcriptional initiation site. GILZ mediates the endogenous and exogenous anti-inflammatory and immunosuppressive effects of GCs in various types of cells, including lymphoid cells, in which it may regulate the activation and apoptosis of cells, which is an important pathophysiological characteristic of the immunopathology of sepsis (6, 51).

GILZ inhibits T lymphocyte activation, apoptosis, and cell proliferation either directly or through antigen-presenting cells (APCs) (52, 53), and inhibit IL-2 withdrawal-induced apoptosis by inhibiting Forkhead Box O3 (FoxO3) transcriptional activity and the proapoptotic gene Bim (54). GILZ also preventsTh-1 and promotes Th-2 differentiation by inhibiting NF-κB activation and nuclear translocation. Transgenic GILZ-overexpressing (GILZ-Tg) mice are less susceptible to Th-1-mediated experimental dinitrobenzene sulfonic acid- (DNBS-) colitis and spinal cord injury. Regulatory T Cells (Tregs) constitute are a subpopulation of T cells that accumulates in the bone marrow and can modulate the immune system, maintain tolerance to self-antigens and control autoimmune disorders. GILZ expression in bone marrow-derived mesenchymal stem cells (BMSCs) and DCs exerts anti-inflammatory effects depending on IL-10-producing Treg and activity. Additionally, GILZ promotes Treg differentiation by activating TGF-β signaling, which is a typical anti-inflammatory factor (55). However, GILZ-KO mice did not have worse collagen-induced arthritis (CIA) compared with WT mice, although the GILZ-KO mice displayed higher T cell proliferation. Furthermore, replenishing GILZ reduces inflammation but does not affect T cell proliferation. Consequently, as a key modulator of Tregs, GILZ plays an anti-inflammatory role in most experimental settings, but the actual role of GILZ in T cells is complex and requires more exploration.

GILZ exerts suppressive effects on B cells by inhibiting cell activation, proliferation, differentiation, apoptosis and IgG production (56). The accumulation of B cell precursors in the bone marrow and peripheral lymphoid organs with elevated Bcl-2 and NF-κB activity has been shown in GILZ-deficient mice (57). The deletion of GILZ increased IFN-γ and AP-1 activity in B cells in a colitis mouse model (58). Furthermore, the above effects were all reversed by the compensation of the GILZ protein, demonstrating the potential therapeutic role of GILZ in regulating B cell-dependent diseases (58).

GILZ not only plays an important role in adaptive immunity, but also inhibits the activity of the innate immune system. In DCs, GILZ and GCs are critical for balancing the anti-inflammatory response and tolerance phenotypes (59, 60), which are closely related to sepsis-induced immunosuppression. GILZ could mediate the downregulation of MHC class II molecules, costimulatory factors, and Treg cell generation (61). Some pro-inflammatory factors including IL-1α, IL-1β, IL-6, and IL-23, were strongly increased in GILZ-KO bone marrow-derived DCs (BMDCs) following upon TLR4 and TLR7 stimulation (62, 63). GILZ prevents DCs from activating the antigen-specific T lymphocyte response. GILZ^-/-^ DCs increased IFN-γ and IL-17 secretion in CD4^+^ T cells and CD8^+^ T cells (64, 65). Moreover, GILZ could regulate antigen capture and cross-presentation by DCs and limits antigen internalization in DCs from GILZ^-/-^ mice (62, 66). Dexamethasone enhanced antigen capture by DCs (67, 68), which does not different from the roles of GILZ. Additionally, GILZ can inhibit DC maturation, which could increase the production of IL-10 and promote the development of tolerant DC phenotypes (65, 69).

The GILZ protein and mRNA levels were obviously decreased in alveolar macrophages (AMs) and THP-1 cells exposed to LPS (6, 46, 70). GILZ could decrease macrophage sensitivity to LPS and proinflammatory cytokines expression (71). GILZ deficiency enhances NF-κB pathway-mediated macrophage phagocytosis. Consistent with this result, GILZ-KO macrophages were observed to have increased NO production (45). Interestingly, GC exerts the opposite effect to promote macrophage phagocytosis (72).

GILZ also inhibits neutrophil migration to inflammatory sites *via* annexin A1 (73) and alleviates the proinflammatory response by inhibiting reactive oxygen species (ROS) generation and the accumulation of leukocytes at the site of inflammation or inducing neutrophil apoptosis (57, 74). Neutrophils derived from GILZ-KO mice showed a stronger capacity to clear pathogens in a candida albicans intraperitoneal infection model. Although the role of GILZ is extensive, it has not been deeply studied in sepsis patients. Furthermore, few studies investigated about the immune response associated with GILZ in myeloid-derived suppressor cells, which perform potent immunosuppressive functions and act on both innate and adaptive immunity.

## GILZ in Sepsis

GILZ has been studied in septic patients and CLP models (**Table 1**). During the hyperinflammatory stage of sepsis, GILZ regulation is contrary to inflammatory release. GILZ mRNA was reduced by 50% in peripheral polymorphonuclear cells from critically ill patients (75, 80). In an experimental model of CLP, the GILZ expression level was also downregulated in both blood cells and the liver after two hours of the CLP procedure (75). Human alveolar macrophages (AMs) were treated with LPS, upon TLR4 activation, both the mRNA and protein levels of GILZ rapidly decreased, while the TNF and IL-6 mRNA levels increased (70, 77).

**Table 1:** A Summary of Preclinical Evidence that revealing the Involvement of glucocorticoid-induced leucine zipper (GILZ) in the pathogenesis of sepsis.

Reference	Mouse Model of sepsis	GILZ-dependent Effects	Treatment
Ballegeer et al. (75)	C57BL/6 and GILZ-Tg mice; CLP induced septic peritonitis.	GILZ-Tg peritoneal leukocytes (CD45+) displayed a significantly higher phagocytic capacity than GILZ-WT cells	**N/A**
Ellouze et al. (76)	C57BL/6 and GILZ-Tg mice; CLP induced septic peritonitis.	Monocytes and macrophages from GILZ-Tg mice showed greater phagocytic capacity and faster bacterial clearance.	**N/A**
Hoppstadter et al. (77)	C57BL/6J and myeloid-specific GILZ knockout (KO) mice; LPS induced endotoxin tolerance.	GILZ-deficient macrophages displayed increased TNF-a and IL-1β expression due to the activation of ERK signaling pathways.	**N/A**
Pinheiro et al. (78)	C57BL/6 and SPRET/Ei mice; LPS-induced lethal inflammation.	Macrophages transfected with TAT-GILZ, containing either the C57BL/6 or SPRET/Ei sequence, showed reduced cytokine production.	TAT-GILZ administration reduced mortality and IL6 serum concentrations
Hang et al. (79)	C57BL/6 mice; LPS-induced septic shock.	Suppression of NF-κB signaling pathways	Short-Chain Alcohols Protect Mice from LPS-induced Septic Shock

The inbred mouse strain SPRET/Ei has been shown to exhibit marked resistance to LPS that depending at least partially on the GR levels and GILZ mRNA was higher than that in C57BL/6 mice (78, 81). In addition, the TAT-GILZ fusion protein, which is a synthetic fusion construct containing the TAT peptide followed by the GILZ cDNA sequence from C57BL/6 and SPRET/Ei, has been shown to reduce mortality [58]. The recombinant GILZ protein was first demonstrated to attenuate colon inflammation in DNBS-induced colitis in 2009 (82). The administration of the TAT-GILZ fusion protein could reduce tissue edema and increase perfusion of the ischemic/reperfused kidney in mice with acute kidney injury (AKI), and improve the disruption of mitochondrial membrane potential and cell death *in vitro* (83, 84). GILZ-Tg mice had a lower mortality and blood bacterial load than GILZ-WT mice in a CLP model (75). Peritoneal leukocytes in GILZ-Tg mice are more likely have a higher phagocytic capacity, which may be mediated in inflammatory resolution due to neutrophils apoptosis or M2−type macrophages polarization (74, 85, 86) In addition, mice with GILZ overexpress mononuclear macrophages (M/M) exhibited an increased survival rate and reduced levels of plasma inflammatory cytokines and blood bacterial load (76). It is likely that overexpression of GILZ elicits an increase in macrophages phagocytosis. A recent study found that the management of short-chain alcohols protects mice from LPS septic shock by increasing peripheral blood GILZ in a dose-dependent manner and suppressing IκB phosphorylation, which is a mechanisms that promoting immune tolerance (16, 79).

Although the upregulation of GILZ can alleviate inflammatory storms and improve survival during the early stage of sepsis, but not endotoxin tolerance (77), the downregulation of GILZ expression independent of GR activation abrogated LPS tolerance and increased responsiveness to LPS by enhancing ERK activity and rescuing MAPK signals, which was independent of GR activation (77). Meanwhile, Wang et al. (87) found that the elevated level of GCs or GILZ is related to late stage inflammation in sepsis, and artesunate can inhibit the upregulation of GILZ mRNA and increase bacterial clearance of hydrocortisone-induced immunosuppression peritoneal macrophages.

Immune phenotypes of infection, especially in sepsis, have many similarities with those in cancer (88). GILZ is a crucial immunosuppressive molecule mediated by GCs in the immunosuppressive tumor environment. Recently, GILZ was shown to be highly expressed in the immunosuppressive tumor microenvironment, which was sufficient to abolish the therapeutic control of tumors (89). DC-specific GILZ deletion or GR antagonists could reverse these negative effects. An online survival analysis suggested that the GILZ level was negatively correlated with prognosis in lung cancer patients (90, 91). These seemingly conflicting data may indicate the cell-specific nature and different stages of the regulation of GILZ in sepsis, which is similar to GCs. As mentioned above, GILZ may play a pivotal role in the immune tolerance of sepsis, although only a few related researches. Of note is, GCs are responsible for osteoporosis though osteoblast formation after long-term GC treatment. In contrast, GILZ increases osteogenic differentiation and inhibits adipocyte formation in mesenchymal stem cells (MSCs) (92). Consistently, cell-specific GILZ overexpression in osteoblasts induced high bone mass and increased bone formation and the osteoblast number (93). Whether GILZ exerts the opposite effect on other GC side effects remains unclear, although the latest published data indicate that GILZ is a critical mediator leading to statin-induced myopathy (90).

Collectively, the potential significance of GILZ in the potential diagnosis, treatment, and prediction of sepsis is worth discussing. To date, few studies investigated the mechanism and therapeutic applications of GILZ in inflammatory diseases and sepsis, and more studies are needed to determine its efficacy and safety.

## Prospects of GILZ in Sepsis

Sepsis and septic shock are both severe diseases that should be treated and resuscitated immediately. Evidence increasingly suggests that the early treatment of sepsis increases the chances of survival, and no study has shown that treatment works better when applied later (94). Although the immune system changes as sepsis progresses, GILZ expression is stable during the late stage of inflammation, especially during immunosuppression, which is probably due to GILZ acting as a downstream molecule and regulation by endogenous cortisol *in vivo.* Hence, GILZ might become a potential monitoring molecule under the regulation of endogenous glucocorticoids or guide treatment with glucocorticoids. Currently, studies are exploring the key role of GILZ in sepsis and whether it can be a critical marker, such as the Rapid Recognition of Corticosteroid Resistant or Sensitive Sepsis (RECORDS) trial (NCT04280497) (95).

According to the current study, GILZ overexpression mice or using GILZ peptides all simulated the effect of early anti-inflammatory agents in acute sepsis. Recombinant GILZ, which couples GILZ to membrane-penetrating sequences, is an attractive prospect, although further studies are needed to demonstrate the feasibility and safety of GILZ in clinical setting. Alternatively, the targeted activation of GILZ expression using the CRISPR/dCas9 activator complex specifically in macrophages could be used (96). It should be noted that a peptide targeting the C-terminal region of GILZ 115-137 aa in the mouse GILZ sequence has demonstrated a potential therapeutic effect in experimental autoimmune encephalitis (EAE) (97), which suggesting that diverse regions of GILZ can bind different partner proteins. It may be necessary to further determine the optional GILZ peptide that binds specific targets.

## Summary

Sepsis is a syndrome associated with an immune system disorder in response to infection. Currently, the mechanism underlying sepsis development is unclear, further limiting treatment. Clinical studies have shown that the earlier sepsis is resolved, the more likely the patient is to survive. GILZ, which is downstream of the GR, has powerful anti-inflammatory effects similar to GCs and might circumvent GCR in sepsis. Proof-of-concept studies have shown that GILZ has significant therapeutic effects in CLP-induced septic models. Additionally, cell-specific targeting of GILZ may circumvent side effects, which could be of particular interest in sepsis models or other experimental models. Cortisol is essential for inflammatory responses, and endogenous and exogenous GCs induce GILZ to change dynamically. Detection of GILZ expression over time can guide the use of GCs and evaluate the timeline of sepsis, which is of great importance for the treatment of sepsis. As a potential alternative treatment to GCs, further research concerning the role of GILZ in sepsis is needed to identify its powerful regulatory role and molecular mechanisms.

## Author Contributions 

Y-JH, J-QX, and YS designed the review. Y-JH and J-QX wrote the manuscript with supervision of YS. All authors contributed to the article and approved the submitted version.

## Funding

This study was supported by National Natural Science Foundation of China (No. 82002026, 81971818, and 81772047).

## Conflict of Interest

The authors declare that the research was conducted in the absence of any commercial or financial relationships that could be construed as a potential conflict of interest.
